# Treatment of Liver Trauma: Operative or Conservative Management

**DOI:** 10.4021/gr2010.02.165w

**Published:** 2010-01-20

**Authors:** Carmen Garcia Bernardo, Josep Fuster, Ernest Bombuy, Santiago Sanchez, Joana Ferrer, Marco Antonio Loera, Josep Marti, Constantino Fondevila, Elizabet Zavala, Juan Carlos Garcia-Valdecasas

**Affiliations:** a Department of Surgery. Hepatic Surgery and Liver Transplant Unit, IMDIM. CIBERHED,IDIBAPS, Spain; b Intensive Care Unit. Department of Anesthesiology. Hospital Clinic, University of Barcelona, Barcelona, Spain

**Keywords:** Liver trauma, Conservative management, Surgical treatment

## Abstract

**Background:**

The liver is one of the most frequently damaged organs when abdominal trauma occurs. Currently, a conservative management constitutes the treatment of choice in patients with hemodynamic stability. The aim of this study is to evaluate the results of an operative and conservative management of 143 patients with liver injury treated in a single institution.

**Methods:**

A retrospective study of the patients admitted with the diagnosis of liver trauma was performed from 1992-2008. The patients were classified according to the intention to treatment: Group I, operative management; Group II, conservative management. Variables analyzed included demographic data, injury classification, associated lesions, surgical treatment, transfusions, morbi-mortality, and hospital stay. We established two periods (1992-1999; 2000-2008) in order to compare diagnosis and management.

**Results:**

A total of 143 patients were analyzed. Thirty-one percent correspond to severe injuries. Conservative treatment was followed in 60.8 % with surgery undertaken in 14.9 % of patients from this group due to failure of conservative treatment. Immediate surgery was carried out in 38.2 %. Total mortality was 14 %. Morbidity (35.7-38.5 %) in the group of immediate surgery and failure of conservative management is similar, but not in mortality (28.6-15.4 %). In the second group (2000-2008) there are more patients with conservative treatment, with a low percentage of failure of this treatment and morbi-mortality.

**Conclusions:**

Conservative treatment is an adequate treatment in a great number of patients. Failure of conservative treatment did not show a higher incidence of complications or mortality but it should be performed in centers with experienced surgeons.

## Introduction

Despite its relatively well protected localization, the liver is the most frequently damaged organ in abdominal injury, although the frequency of splenic lesions is greater in non penetrating trauma [1]. In Europe in the last 10 years the incidence of liver trauma appears to have risen due to the increase in the frequency of abdominal contusions because of traffic accidents [2, 3].

During the last decade there has been a change in the therapeutic protocols related to liver trauma, with many studies having been published in the literature [4-7]. Surgery is no longer the only option available. Despite the initial scepticisim there has been a progressive acceptation of non surgical treatment, imitating the experience of the pediatric surgeons [8, 9], with the aim of obtaining a reduction in morbi-mortality. Surgery has been reserved for extensive lesions with condition of hemodynamic instability or for the treatment of the complications. Surgical technique has also evolved towards limited resection-debridement, selective vascular ligation and the use of perihepatic packing [2-4].

The objective is to achieve a reduction in the mortality and the rate of complications. In mild trauma this appears to have been achieved, however, this is difficult to do in extensive injuries with vascular involvement.

The aim of this study was to analyze the effectiveness and the morbi-mortality of both conservative and surgical treatment in a series of patients with hepatic injury attended in our instutition.

## Patients and Methods

We herein review our experience in the treatment of liver trauma in adults over seventeen years (1992 - 2008) including all the patients diagnosed with hepatic injury reported in the registry of admittance to the Emergency Department of our Institution. The liver trauma was classified according to the Hepatic Injury Scale (HIS) of the American Association for the Surgery of Trauma [10].

To analyze the results the patients were divided into two groups, Group I: operative treatment; Group II: conservative treatment. Secondarily, to evaluate the impact and optimization of diagnosis and treatment, we stratified our patients into 2 groups by time period: 1992-1999 and 2000-2008.

The decision as to which treatment to apply depended on the surgeon, with conservative treatment being implemented in patients fulfilling the following criteria: a) hemodynamic stability or correct response to plasma volume expansion; b) transfusion requirements related to hepatic injuries of less than 2-3 red blood cell concentrates; c) absence of signs of diffuse peritonitis on physical exploration; and d) no suspicion of injuries associated with abdominal surgery on imaging tests. The initial radiological exploration was carried out with ultrasonography or abdominal computerized tomography (CT) scan according to what was available at that time. This group of patients remained under strict clinical control, hemodynamic monitorization, and seried determination of hemoglobin and absolute bed rest for a period of 48 - 72 hours. The appearance of hemodynamic instability, clinical signs of peritonism and/or a continued reduction in hematocrit values was considered as non surgical treatment failure with surgery being thereby indicated. On confirmation of the absence of clinical changes and if the associated injuries so permitted, the patients were transferred to conventional hospitalization wards. Abdominal CT was routinely performed prior to hospital discharge and was repeated after 2 - 3 months to verify the resolution of the injuries and to authorize complete renewal of daily activities.

Patients who did not fulfill any of the previously mentioned conditions were evaluated for immediate surgical treatment. Surgeons specialized in hepatic surgery or surgeons under their supervision undertook emergency surgery.

The variables analyzed for the two groups of patients included demographic variables, classification of hepatic injury, associated lesions, surgical technique, transfusion requirements, hospital stay and morbi-mortality.

## Results

From April, 1992 to October, 2008, 143 patients (79.7% males) with liver trauma were treated in our center. The mean age of the patients was of 32 ± 14.7 years (range 16 - 82 years). The injuries were due to traffic accidents (63.3%), stab wounds (10.5 %), falls (11.2%) and firearms (2.1%). Associated abdominal lesions were presented in 41.3 % of the cases: kidney (26-18.2%), spleen (20-13.9%), diaphragm (6-4.2%), colon (3-2.1%), small intestine (2-1.4%) and others lesions (gallbladder, stomach) (6-4.2%). A total of 74.8% of the patients had presented extraabdominal lesions: thoracic injury (79-55.2%), bone fractures (60-41.9%), cranioencephalic trauma (34-23.8%), pelvic (14-9.8%) and vertebral lesion (9-6.3%).

The mean Injury Severity Score (ISS) [11] was of 25.8 ± 12.1 points (range 4 - 54). The classification of the severity of the hepatic injuries according to the HIS criteria was as follows, grade I: 23 cases (16.1%); grade II: 34 cases (23.8%); grade III: 56 cases (39.2%); grade IV: 19 cases (13.3%); and grade V: 11 cases (7.7%).

### Surgical treatment

Fifty-six patients (39.2%) underwent surgery on admission due to hemodynamic instability (71.4 %). Other causes for surgical treatment were: signs of peritoneal irritation on physical exploration, pneumoperitoneum, suspicion of diaphragmatic injury, renal injury and grade V radiologically diagnosed hepatic injury. Ten patients with grade V had hemodynamic instability and required more than 10 red blood cell concentrates. Eight of the 17 cases who underwent surgery with grade I - II hepatic injury according to HIS classification presented associated lesions which led to surgery (4 splenic, 3 retroperitoneal hematomas, 1 gastrosplenic short vessel lesions).

The surgical techniques performed included vascular suture in 21 cases (37.5%, including18 simple sutures, 2 right hepatic and 1 porta veins reconstructions); exploratory laparotomy in 12 (21.4%); hepatic resection in 10 (17.8%) patients with 8 right hepatectomies; packing in 7 (12.5%); electrocoagulation in 5 (8.9%) and procedures not related to the liver in 19 patients (33.9%). Patients with packing had 2 avulsions and 5 lacerations, localized in right liver, all received more than 10 red blood cell concentrates. There were 4 deaths during the exploratory laparotomies. Complications were presented in 20 patients, 18 (32.1%) related to surgery and five respiratory complications (8.9%). In the group receiving surgical treatment (excluding the 12 cases because of death during the first 48 hours), the complications were: biliary leaks in 7 cases (10.1%); wound infection 4 (5.7%) and intrabdominal abscess 2 (2.9%). Others were hemoperitoneum (one patient), ileal perforation (one patient), pseudoaneurism of renal artery (one patient), evisceration (one patient) and empyema (one patient). Ten patients were reoperated (17.5%), three for persistent biliary fistula; three to remove packing; one hemoperitoneum due to retroperitoneal hemorrhage; one ileal perforation which had not been previously observed; one peritonitis and one for intrabdominal hiperpressure. In three cases with biliary fistula before surgery we performed two endoscopic retrograde cholangiopancreatography and one percutaneous transhepatic drainage. Red blood cell transfusion was required in 51 cases (91.1%) (mean 10.8 ± 10.7 red blood cell concentrates). Sixteen patients in this group died (28.6%) (Table 1), 11 were due to causes directly related to the hepatic injury during the first 48 hours. The mean hospital stay in this group was of 20.4 ± 22.3 days (Table 2).

**Table 1:** Distribution of Mortality by Type of Initial Treatment (Operative, Conservative and Failure) and Grade of the Injury

Grade	Operative		Conservative		Failure	Total Mortality
	N (%)	Mortality*	N (%)	Mortality*		N (%)†
I (n = 23)	5 (21.7)	-	18 (78.3)	-	-	-
II (n = 34)	12 (35.3)	3 (25)	22 (64.7)	2 (9.1)	1 (4.5)#	6 (14.7)
III (n = 56)	22 (39.3)	3 (13.6)	34 (60.7)	-	6 (17.6)	3 (5.3)
IV (n = 19)	8 (42.1)	4 (50)	11 (57.9)	1 (9.1)	4 (36.4)	5 (26.3)
V (n = 11)	9 (81.8)	6 (66.6)	2 (18.2)	1 (50)	2 (100)#	8 (63.6)
Total:	56** (39.2)	16 (28.5)	87** (60.8)	4 (4.5)	13**	22 (15.3)

* Percentage of mortality by initial treatment group and grade of injury

† Percentage of mortality by grade of injury.

** Number total of patients by group (operative, conservative and failure)

# one patient in each group (grade II and V) died in conservative treatment failure

**Table 2:** Summary of Outcome by Treatment Group

	Operative	Conservative†	Total	Failure
	N=56 (%)	N=87 (%)	N=143 (%)	N=13 (%)
Morbidity				
YES	20 (35.7)	10 (11.5)	30 (20.9)	5 (38.5)
NO	36 (64.3)	77 (88.5)	113 (79.1)	8 (61.5)
Mortality				
YES	16 (28.6)*	4 (4.5)	20 (13.9)*	2 (15.4)
NO	40 (71.4)	83 (95.5)	123 (86.1)	11 (84.6)
Stay	20.4 ± 22.3	15.2 ± 12.9	17.3 ± 17.1	16.1 ± 9.5

* 12 patients died during the first 48 hours.

† On data analysis of the conservative group, the group of failure of conservative treatment was included.

### Conservative treatment

Eighty-seven patients (60.8%) initially received conservative treatment that was effective in 74 (85%) cases. The morbidity in this group was 6.8% (5 cases).

Complications on the patients who did well with the conservative treatment were a respiratory infection, one adult respiratory distress syndrome (ARDS) and one paralytic ileum. The mortality in this group was of 4 patients (Table 1). One died as a consequence of associated severe cranioencephalic trauma, one for multiorgan failure and two patients died after failure of conservative treatment. The mean hospital stay of this group was of 15.2 days (range 5 - 90 days). The global rate of transfusion requirements in the non surgical treatment group was of 31.1% (mean 5.5 ± 7.5 red blood cell concentrates). Three patients with conservative treatment underwent embolization: splenic, pudendal and hepatic artery.

### Failure of conservative treatment

In 13 patients (9.1%), non surgical treatment failed with surgery being required (Table 1). The reason for failure was hemodynamic instability in 11 cases and a maintained low hematocrit values in two cases. For control we used abdominal echography in 4 patients and CT in other four. One patient required embolization of hepatic artery and surgery because bleeding continued after the embolization.

**Figure 1:**
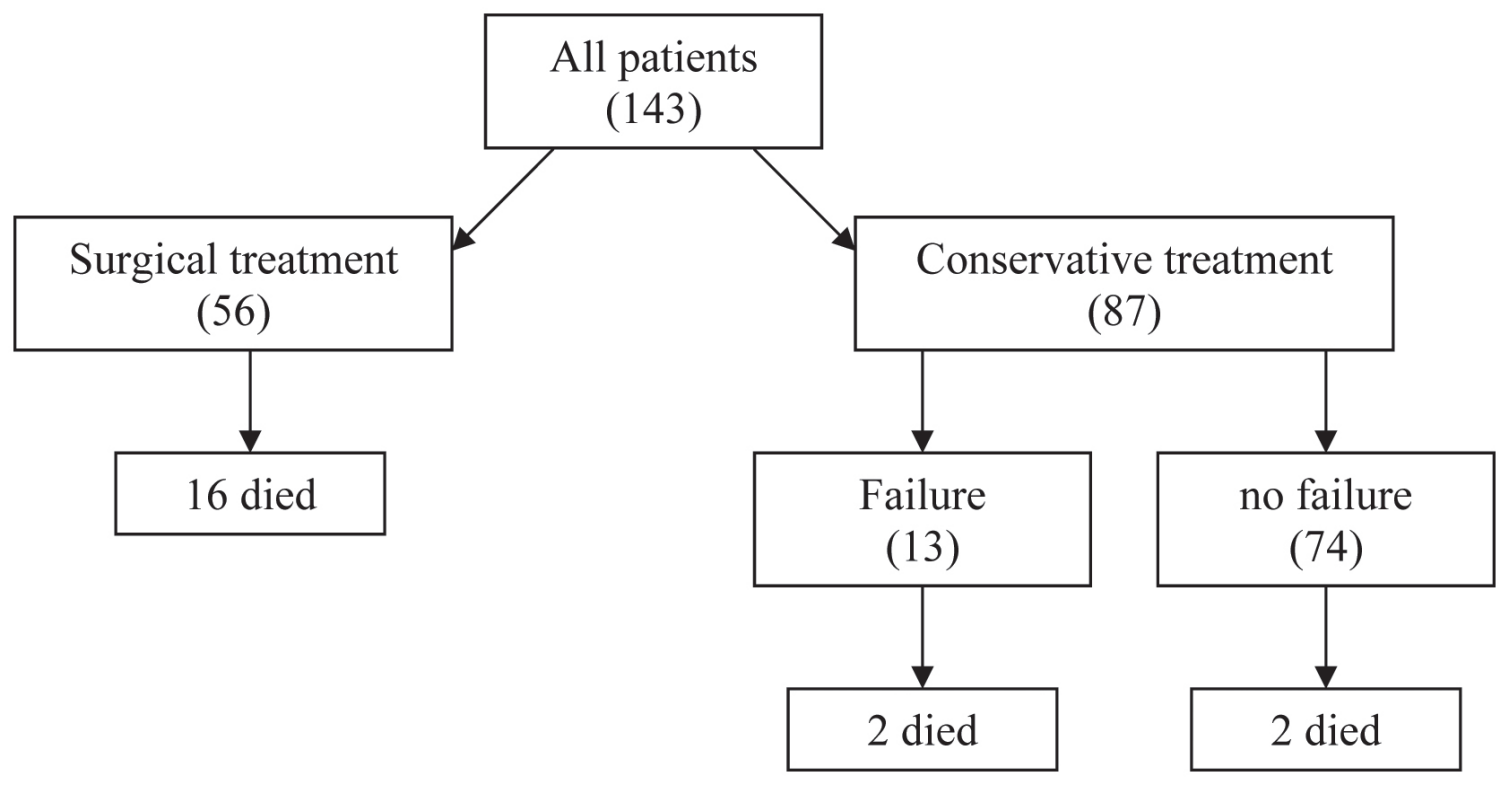
Management and mortality.

Nine of the patients were underdiagnosed after undergoing the complementary explorations, with grade V hepatic injuries going undiagnosed in two cases. Likewise, 4 splenic lesions were not diagnosed leading to reintervention in 3 cases, with hemorrhage from the hepatic injury not being observed and one right diaphragmatic injury was also not observed. Eleven underwent surgery during the first 24 hours and the remaining two cases had surgery on the 4th and 5th day, respectively. Two patients died (15.4%), due to ARDS in one patient with severe cranioencephalic trauma, and the other death was due to intrahospitalary pneumonia with multiorgan failure. The following complications were presented: one biliary leak, one bleeding, one respiratory distress and two respiratory infections. The mean hospital stay was of 16.1 days (range: 7 - 38 days). Blood transfusion was required in 92.3% of the patients (mean 13.3 ± 10.4 red blood cell units). Figure 1 shows the management and mortality and Table 2 summarizes the morbid-mortality according to treatment group.

The uni- and multi-variant analysis were performed. Compared with the patients who underwent conservative management, patients who underwent a surgical treatment had a higher initial and final injury grade, more morbidity, mortality, hepatic mortality, higher injury severity score (ISS), a more use of packed red blood cell (RBC), fresh frozen plasma (FFP), Platelet (PLT) (Table 3). In multiple logistic regression model, only initial and final injury grade are predictives factors (Table 3).

**Table 3:** Risk factors for treatment by Uni-multivariante Analysis

Univariante Analysis			
Variable	Final conservative management (n =74)	Final surgical treatment (n = 69)	P
Initial injury grade			< 0.0001
No injury	1	32	
I	17	4	
II	22	5	
III	27	20	
IV	7	6	
V	0	2	
Final injury grade			< 0.0001
I	18	5	
II	21	13	
III	28	28	
IV	7	12	
V	0	11	
Morbidity			< 0.0001
Yes	5	25	
No	69	44	
Mortality			< 0.0001
Yes	2	18	
No	72	51	
Hepatic Mortality			< 0.0001
Yes	1	13	
No	73	56	
ISS *	23.1 ± 15	30.1 ± 13.1	< 0.0001
RBC *	5.2 ± 7.5	11.2 ± 10.6	0.002
FFP *	0.4 ± 1.6	2.6 ± 3.1	< 0.0001
PLT *	0.02 ± 0.1	0.5 ± 1.1	< 0.0001

Multivariante Analysis (Multiple Logistic Regression Model)				
Variable	p	OR	95% CI	
Inicial grade injury	< 0.0001	3.8	2.05	7.08
Final grade injury	< 0.0001	0.2	0.08	0.4

* Values expressed as mean ± standard deviation. Abbreviations: ISS: injury severity score; RBC: red blood cell; FFP: fresh frozen plasma; PLT: Platelet, OR: odds ratio; CI: confidence interval.

Table 4 presents the risk factors for injury grade identified by univariable analysis: hemodynamic inestability, vascular injury, surgical technique, pringle, mortality, hepatic mortality, hemoperitoneum, lesion size, red blood cell (RBC), fresh frozen plasma (FFP), Platelet (PLT), hospital stay. However, only hemoperitoneum and lesion size are predictives factors by multivariable analysis (Table 4).

**Table 4:** Comparison of Patients With Low and High Injury Grade

Univariante Analysis			
Variable	Grade I-II-III	Grade IV-V	P
Hemodynamic instability			0.008
Yes	23	19	
No	23	4	
Vascular injury			0.001
Yes	0	7	
No	46	16	
Surgical technique			< 0.0001
Exploratory laparotomy	13	1	
Packing	0	8	
Vascular suture	20	5	
Electrocoagulation	6	0	
Hepatic resection	6	9	
Pringle			< 0.0001
Yes	4	15	
No	42	8	
Mortality			0.005
Yes	7	11	
No	39	12	
Hepatic mortality			< 0.0001
Yes	3	10	
No	43	13	
Hemoperitoneum*	1377 ± 897	2939 ± 1351	< 0.0001
Lesion size *	5.1 ± 2.2	9 ± 2.8	< 0.0001
RBC *	6.2 ± 6.5	18.7 ± 12.3	< 0.0001
FFP *	.1.1 ± 2.3	3.03 ± 3	< 0.0001
PLT *	0.07 ± 0.3	1.1 ± 1.9	< 0.0001
Hospital Stay	18.9 ± 18.1	11.4 ± 10.7	0.003

Multivariante Analysis (Multiple Logistic Regression Model)				
Variable	p	OR	95 % CI	
Hemoperitoneum	0.02	1	1	1
Lesion size	0.013	2.8	1.2	6.4

* Values expressed as mean ± standard desviation

Abbreviations: RBC: red blood cell; FFP: fresh frozen plasma; PLT: Platelet, OR: odds ratio; CI: confidence interval

### Comparative of two periods (1992-1999; 2000-2008)

Epidemiology, clinic, treatment characteristics and complications in both groups show in Table 5. We observed that in the second period there was a high number of patients who were operated for inestability (82.3% vs 66%, p = 0.746).

**Table 5:** Comparison of Patients in Two Periods

Variable	1992-1999 N = 88 (%)	2000-2008 N = 55 (%)	P
Age (yrs)*	30.8 ± 13	33.9 ± 17.1	0.441
Male sex	68 (77.3)	46 (83.6)	0.242
Hospital stay*	15.1 ± 13.4	20.8 ± 21.3	0.071
ISS*	25.8 ± 12.2	25.8 ± 11.8	0.896
Etiology			0.038
Traffic accidents	60 (68.2)	45 (81.8)	
Stab wounds	14 (15.9)	1 (1.8)	
Falls	10 (11.4)	6 (10.9)	
Firearms	3 (3.4)	0 (0)	
Others	1 (1.1)	3 (5.5)	
Diagnostic technique			< 0.0001
No	8 (9.1)	0 (0)	
Ultrasound	72 (81.8)	16 (29.1)	
TC	4 (4.55)	39 (70.9)	
DPL*	4 (4.55)	0 (0)	
Injury grade			0.022
I	8 (9.1)	15 (27.3)	
II	20 (22.7)	14 (25.5)	
III	40 (45.5)	16 (29.1)	
IV	11 (12.5)	8 (14.5)	
V	9 (10.2)	2 (3.6)	
Abdominal injuries			0.092
Yes	32 (36.4)	27 (49.1)	
No	56 (63.6)	28 (50.9)	
Extrabdominal injuries			0.041
Yes	61 (69.3)	9 (16.4)	
No	27 (30.7)	46 (83.6)	
Hemodynamic inestability	31 (35.2)	15 (27.3)	0.211
Treatment			0.077
Nonoperative management	49 (55.7)	38 (69.1)	
Surgery	39 (44.3)	17 (30.9)	
Surgical technique			0.087
Exploratory laparotomy	9 (10.2)	5 (9.1)	
Packing	3 (3.4)	5 (9.1)	
Vascular suture	21 (23.8)	4 (7.3)	
Electrocoagulation	5 (5.7)	1 (1.8)	
Hepatic resection	6 (6.8)	9 (16.4)	
Failure of conservative treatment	8 (16.3)	5 (13.1)	0.482
Morbidity			0.015
Yes	24 (27.3)	6 (10.9)	
No	64 (72.7)	49 (89.1)	
Mortality			0.532
Yes	12 (13.6)	8 (14.5)	
No	76 (86.4)	47 (85.5)	
Hepatic Mortality			0.534
Yes	9 (10.2)	5 (9.1)	
No	79 (89.8)	50 (90.9)	
RBC *	7.6 ± 8.2	13.7 ± 12.3	0.033
FFP *	1.3 ± 2.6	2.4 ± 2.6	0.003
PLT *	0.3 ± 1.9	0.8 ± 0.9	< 0.0001

* Values expressed as mean ± standard desviation

Abbreviations: ISS: injury severity score; DPL: diagnostic peritoneal lavage; RBC: red blood cell; FFP: fresh frozen plasma; PLT: Platelet

In the last years, CT is the principal study for diagnosis of liver injury instead of ultrasound used in the first period (p < 0.0001). In the first period the patients had more high-grade injuries (III-V) (68.2% vs 47.2%, p = 0.022) and extrabdominal lesions (69.3% vs 16.4%, p = 0.041) respect to the second period. Conservative management is the most used in the last years (69.09% vs 55.68%, p = 0.077), with surgical techniques more aggressive: 9 hepatic reseccion vs 6 in the first period; 4 simple suture vs 21 and 5 exploratory laparotomy vs 9 (p = 0.087). Failure of conservative treatment in first period is higher than in the second (16.3% vs 13.1%, p = 0.482).

Mortality was similar in both periods of the study (13.6% in the first vs 14.5% in the second, p = 0.532). Morbidity related to surgery and medical complications decreased since 2000, surgical (38.4% vs 29.4%, p = 0.369) and medical (14.7% vs 3.6%, p = 0.028). General morbidity decreased in the second period (p = 0.015).

## Discussion

In the last 15 years, the treatment of liver trauma has progressively evolved [4, 12]. At the beginning of the 1990’s several articles reported the possibility of non surgical treatment in patients with hemodynamic stability similar to what is carried out by pediatric surgeons in cases of hepatic-splenic injuries [9, 12]. The aim of this type of treatment is to thereby not only decrease the number of non therapeutic laparotomies [13-15] but also to achieve a reduction in the values of morbi-mortality. In this group of patients immediate surgery is substituted by initial non surgical treatment with close patient supervision. Surgery is indicated in cases of continued hemorrhage or the suspicion of the presence of determined associated lesions. Fortunately, a high percentage of injuries, around 85 %, are not severe (HIS < grade IV) [4, 16], which previously were treated with electrocoagulation, topical hemostatic agents or superficial ligature. In these injuries the hemorrhage had ceased at the time of surgery in a considerable number of cases [14]. It is in this group of patients that conservative treatment undoubtedly achieves the greatest percentage of success. However, in the remaining 10% - 20% of the severe hepatic injuries the decision as to whether surgery is necessary represents a difficult challenge for the surgeon.

Therapeutic evolution has become possible thanks to the diffusion of imaging techniques such as echography and abdominal CT which are more rapid, sensitive and specific in the diagnosis of abdominal injuries [2, 12, 14, 17, 18], and they have replaced peritoneal lavage because of its low specificity and bad prediction of the need for laparotomy [17], despite its high sensitivity and speed of application.

In our center we routinely use abdominal echography as the first complementary exploration in the study of abdominal trauma. If the patient presents signs of hemodynamic instability, echography is immediately performed with portable equipment in the Emergency Department. This is a cheap, non-invasive exploration which is rapid and has a high sensitivity and specificity of 80% - 95% [2, 19], for the detection of intraabdominal injuries, although it is a technician-dependent exploration with little specificity for detecting visceral lesions. With the presence of findings leading to suspicion of hepatic injury in a stable patient, the study is completed with abdominal CT with endovenous contrast to provide better knowledge of the liver injury, HIS classification and the determination or discarding of associated intraabdominal injuries. Up to three years ago only echographic study was frequently performed in patients with mild injuries which led to underevaluation of hepatic injury and the missing of other lesions which posteriorly caused complications. Although the initial treatment would have changed in few patients, we believe that an abdominal CT with contrast should be carried out within the first 24 hours on suspicion of hepatic injury. CT scanning has become the gold standard for diagnosis of solid organ injury and allows reasonably accurate grading of organ injuries and provides crude quantitation of the degree of hemoperitoneum [12].

In the series published, the applicability of conservative treatment in patients with liver injury has varied from 35% to 82% [6, 16] according to the year, the selection criteria and the number of patients studied. The two main variables guiding the therapeutic approach were hemodynamic instability and the need for transfusion [9, 20, 21]. In our center conservative treatment was implemented in 60.8 % of the cases in the last 17 years with a failure rate of 15%, which is slightly higher than what has been reported in the literature [6, 16].

There are no predictive criteria to allow either the selection of the type of adequate treatment or to predict the failure of conservative treatment. Thus, the application of conservative treatment in cases of liver trauma obliges the surgeon to perform continuous monitorization of the patient during the first 48 hours and to have adequate infrastructure to allow immediate surgery on observation of clinical deterioration of the patient [7]. During the first years most series limited the cases to non-severe injury (grade ≤ III) [5], restricting the use of conservative treatment to values below 40% of the cases. Posteriorly, the good results achieved led to progressive widening of the inclusion criteria [14].

Feliciano et al proposed conservative treatment for any lesion regardless of the magnitude as long as the patient remained hemodynamically stable and with hemoperitoneum of less than 500 ml as estimated by CT scan [22]. Currently most authors consider that the decisive factor in deciding the implementation of conservative treatment should be hemodynamic stability after initial recovery independently of the grade of the injury and the quantity of hemoperitoneum estimated by CT [2, 15, 20]. In the present series all the patients with grade V injury underwent surgery. In two cases conservative treatment was implemented but failed due to hemodynamic instability. In our limited experience severe grade V injuries appear to be a predictive factor requiring surgical treatment. Nonetheless, in a series of 500 patients who received conservative treatment, Malhota et al described a failure rate of only 23 % in the group of patients (n = 30) with grade V lesions [16]. Other series show that nonoperative management of high-grade liver injuries have been successful [14] but is associated with significant morbidity and correlates with the grade of liver injury [23]. Complications require a multidisciplinary treatment and a strategy should be anticipated in grade IV and V injury [24]. High-grade injuries can be managed nonoperatively, if operative intervention is not required for hemodynamic instability or associated injuries, with a low mortality [4, 14, 15, ].

In this subgroup with high risk of conservative treatment failure, the use of angiography with selective embolization of the hepatic injuries may be useful [4, ]. In our series only one case has been treated with selective embolization of hepatic artery. The main cause of the low use of angiography is that the majority of vascular injuries are venous [31]. The mortality from juxtahepatic venous injuries is generally reported from 50% to 80% and the direct approach is the correct attitude in these lesions [32]. It is important to emphasize that in our series the indexes of morbi-mortality were not greater in the patients with conservative treatment failure compared to similar injuries in the surgical group with the values of both groups being similar to those reported by other groups [16, ].

Our comparative study between the two groups shows a development in diagnosis and similar treatment displayed in the others papers [4, 12] but in first period the patients had hepatic and extrabdominal lesions more heavy. The use of CT as gold standard technique in diagnosis and the conservative treatment in stable patients with low consumption of blood products and even in high grade injuries (IV-V) are the principals conclusions in this and others multiple reports [2, 14, 15, 20, 25].

In summary, conservative treatment of hepatic injury is applicable (83.1%) in patients presenting hemodynamic stability, although in grade V injuries there is a high risk of conservative treatment failure and, in our opinion, these patients should undergo surgical treatment after diagnosis. Failure of conservative treatment does not necessarily lead to an increase in the incidence of complications or mortality in centers with adequate infrastructure with monitorization and/or continued intensive therapy and the immediate possibility of performing surgery.

## Abbreviations

HIS: hepatic injury scale
CT: computerized tomography
ISS: injury severity score
ARDS: adult respiratory distress syndrome
RBC: red blood cell
FFP: fresh frozen plasma
PLT: platelet
OR: odds ratio
CI: confidence interval
DPL: diagnostic peritoneal lavage
